# Defensive medicine and cesarean sections in Brazil

**DOI:** 10.1097/MD.0000000000024176

**Published:** 2021-01-08

**Authors:** Edson Luciano Rudey, Maria do Carmo Leal, Guilhermina Rego

**Affiliations:** a School of Medicine, Porto University, Porto, Portugal; b Oswaldo Cruz Foundation (FIOCRUZ), Rio de Janeiro, Brazil.

**Keywords:** bioethics, Brazil, cesarean section, defensive medicine, obstetric litigation, questionnaire

## Abstract

Brazil has a high rate of cesarean sections (CS) that cannot be solely justified by women's clinical conditions; thus, other causes, for example, CS on maternal request and physicians’ fear of litigation as possible influencing factors, cannot be overlooked.

This study aimed to identify through a survey whether Brazilian gynecologists and obstetricians (GOs) perform defensive CS.

In this cross-sectional, descriptive study, a questionnaire was administered. The target population comprised of GOs who were members of premier Brazilian professional associations of gynecology and obstetrics. A total of 403 GOs participated in the survey using an obstetrics questionnaire about litigation and defensive medicine (DM). Statistical analyses were performed on pairs of variables to determine the risk factors of performing CS due to concerns of complications during vaginal delivery and to avoid lawsuits.

The mean age of the GOs was 47.7 years who were mostly female (58.3%) and having worked professionally in both public and private sectors (71.7%). Of all participants, 80.6% had been sued or knew an obstetrician who had been sued. The obstetricians who had been sued or who knew a colleague that had been sued exhibited a significantly higher likelihood of performing defensive CS than physicians who had not been sued or did not know physicians who had been sued. The perception of a higher risk of lawsuits against obstetricians influenced the practice of DM and led to a more than six-fold increase in CSs in specialists with this perception compared to specialists who did not believe the presence of an increased risk of litigation in obstetrics existed.

The majority of Brazilian GOs perform defensive CS. It is important to consider DM as one of the causes of high CS rates in Brazil and include it in the development of public policies to reduce these CS rates.

## 1 Introduction

Medically indicated cesarean sections (CS) can prevent maternal and neonatal mortality^[1,2]^; however, similar to any other surgery, there are associated health risks for both women and children.^[3–5]^ In recent decades, CS rates have increased worldwide, having nearly doubled since 2000,^[6–10]^ and its prevalence varies by region, as well as along economic and cultural lines. In Latin America and the Caribbean, the CS rate is 10 times higher than in certain regions of Africa.^[9]^ In Brazil, CS rates have notably increased among educated women, even for low-risk pregnancies.^[10]^

Increased CS rates are partially attributable to an increase in the number of nulliparous women who become pregnant at an older age and the increasing prevalence of obesity.^[1]^ Despite changes in population characteristics, the results of the “Being Born in Brazil” (Nascer no Brazil) survey showed that there is no clinical justification for such a high CS rate.^[11]^ These rates may have been influenced by other economic and sociocultural factors, such as the increased perception that women should claim their right to reproductive self-determination (CS on maternal request), childbirth care models that make CS a more convenient option for Gynecologists and obstetricians (GOs), as well as physicians’ fear of litigation.^[7,11–16]^

GOs are among the medical professionals with high litigation risk^[17,18,19]^ thereby resulting in higher lawsuit settlements.^[19,20]^ In China, more than 60% of GOs have been involved in at least 1 work-related lawsuit.^[21]^ Relevant data indicate an increase in litigation cases in the obstetrics area, which leads to changes in the medical conduct of GOs and engenders dissatisfaction with their profession.^[22]^ Yvonne et al reported that, compared with those who have not been sued, GOs who were part of an obstetrics lawsuit were increasingly likely to suggest a CS,^[23]^ indicating that CSs are not always performed according to patient need, but as a defensive method to avoid potential litigation.

Defensive medicine (DM) is defined as a practice wherein a healthcare professional makes decisions out of fear of litigation and not for the benefit of the patients.^[24–26]^ DM can be positive when additional procedures are performed without proven necessity and negative when high-risk patients and procedures are avoided.^[27]^ These defensive behaviors have been questioned both morally and ethically^[28–30]^ because they can increase not only the costs of health services,^[29,31]^ but also patient's risk.^[31,32]^

In North America, GOs practice significantly more DM than medical professionals in other areas. GOs avoid performing procedures and interventions that pose a high risk of litigation, such as vaginal births and vaginal births after a CS.^[17]^ Defensive CSs without any medical indication have been performed in other countries^[32,33]^ out of fear of litigation stemming from normal childbirth care.^[34]^ Therefore, when discussing CS rates, examining obstetricians’ perspectives is crucial, as they play a key role in selecting the birth method. Furthermore, focusing on their opinions regarding the subject is important for successful policy implementation in this scenario.^[10,34]^

Brazil has high CS rates,^[10]^ and for its reduction, great efforts are required to ensure that a CS is only performed when necessary,^[35]^ or at least only when requested by the pregnant woman. Incentives for exploring methods to reduce CS rates are frequently improved.^[36]^ This includes the avoidance of a CS as the first method of delivery in nulliparous women as this may help reduce the high rates of CSs in Brazil.^[37]^ Interventions outlined by the Cochrane Collaboration to reduce CS rates involve policies that limit legal liability against physicians; however, this type of intervention requires further research to be fully understood.^[38]^ In Brazil, lawsuits against physicians have increased by 1,600% compared with that in the previous decade, and this is a crucial concern.^[39]^ Therefore, understanding the relationship between DM and CS rates is essential to address this issue. However, despite the relevance of the subject, to the best of the authors’ knowledge, no study has been conducted on this matter in Brazil. Therefore, this study aimed to identify through a survey whether Brazilian gynecologists and obstetricians (GOs) perform defensive CS.

## 2 Methods

### 2.1 Study design and population

In this cross-sectional, descriptive study, a questionnaire was administered. The target population comprised GOs who were members of premier Brazilian professional associations of gynecology and obstetrics. We sent a request to all state associations affiliated with the premier Brazilian gynecology and obstetrics associations to invite their associate physicians to participate in the survey or provide us with contact information of Brazilian GOs. Participation was voluntary and depended on the rules of each association, and for this reason some associations did not participate.

This study was approved by the research ethics committee through the unified national database of research records, called Brazil Platform (Plataforma Brazil, number 3,258,050). The invitation developed by the authors contained text that clearly and succinctly explained the purpose, motivation, and importance of the study as well as contained a link to the online questionnaire survey. In addition, anonymity of the participants was guaranteed. The physicians received information about the researchers and were told how to contact them if they needed more information. All physicians who responded to the questionnaire provided informed consent before completing the survey.

Based on a power analysis calculation, a sample size of 380 would be statistically representative of the population, with 95% confidence and an error measure of 5%. The survey was open from October 2019 to January 2020. Only complete questionnaires (n = 403) were accepted for sampling. We were unable to ascertain the non-response rate since we do not have the exact number of GOs who received an invitation with the link to the questionnaire.

### 2.2 Design

The questionnaire was developed based on 2 surveys from previous studies that were considered valid,^[40–42]^ which were presented by Studdert et al^[17]^ and the Congress of the United States Office of Technology Assessment.^[43]^ Furthermore, a similar research questionnaire was used in a study involving GOs in Turkey,^[34]^ and the clinical experience of researchers was considered. Necessary measures were taken to translate the questionnaires into Portuguese (Supplementary File 1).

The questionnaire was initially evaluated for quality, relevance, and clarity by 2 physicians (i.e., GO specialist judges), who were asked to edit the questionnaire and suggest improvements. The prerequisites for participation as a specialist judge were status as a professor at a university and ≥10 years of experience in both professional medical practice and scientific research. All objective questions and answers in the questionnaire went through an objective examination. After the evaluation, the specialist judge checked one of the available options:

(a) “I attest that all questions and answers are of adequate quality, relevance, and clarity”;
(b) “I attest that almost all questions and answers are of adequate quality, relevance, and clarity”;
(c) “I attest that almost none of the questions and answers are of adequate quality, relevance, and clarity”;
(d) “I attest that none of the questions and answers are of adequate quality, relevance, and clarity.”

Following this evaluation, the authors made the necessary adaptations and evaluated the instrument via a pilot project involving 13 GOs, who answered the questionnaire to re-evaluate potential flaws and necessary corrections. The questionnaires in the pilot study were not included in the sample.

The goal of the questionnaire was to collect demographic and occupational data, thereby evaluating the physicians’ perceptions regarding professional litigation within their specialty and potential DM practices within obstetrics. The estimated time for each physician to complete the questionnaire was 5 minutes. Most of the questions were close-ended, and the format varied according to question type (i.e., binary questions with a “yes” or “no” answer, or multiple-choice) using a Likert scale.

Variables included age, sex, years of experience as a physician, workplace (public and/or private sector), having or not having civil liability insurance, personal perspectives regarding medical litigations, and the use of defensive medical practices related to birth methods. GOs who had been sued or who knew a colleague who had been sued were included in the same category. The authors chose not to separate such cases into different variables, since DM is defined as a practice wherein a healthcare professional makes decisions out of fear of litigation.^[24–26]^ Furthermore, the fear of litigation that DM generates does not stem solely from being previously sued.^[26]^ The aim of the study was not to investigate the results of the lawsuits faced by doctors, their subsequent behavior or to investigate the probable compensation paid.

### 2.3 Statistical analysis

Statistical associations among the variables were evaluated using the Fisher exact test, with odds ratios and 95% confidence intervals as association measures. Statistical analyses were performed on pairs of variables to determine the risk factors of performing CS out of fear of complications involving vaginal delivery. This was done to assess the intention of GOs to avoid litigation according to certain demographic characteristics, such as sex and length of professional experience; these demographic characteristics were statistically tested in a previous study on DM.^[17]^ Moreover, we performed statistical analyses to determine the risk factors of performing CS for variables, such as being sued for “medical error,” or knowing an obstetrician who has been sued, and the perception regarding the risk of ligation in obstetrics, compared with that of other medical specialties. These variables were selected because they are potential causes of DM. A test for comparisons between proportions was used to compare the frequency of certain practices in Brazil and in other countries. The significance level was set at 5%, and statistical environment R (R Development Core Team) version 3.3.1 was used for all analyses.

## 3 Results

Only complete questionnaires (418 responses) were accepted. The 15 questionnaires answered by the evaluating physicians (n = 2), as well as the drafts used in the pilot study (n = 13), were excluded from the sample. The final sample consisted of 403 completed questionnaires.

The mean age of the GOs who responded to the questionnaire was 47.7 years. The majority were female (58.3%) and worked professionally in both public and private sectors (71.7%). Table 1 shows the demographic data and professional characteristics of the respondents. Tables 2 and 3 show responses to medical litigation and to DM in obstetrics.

**Table 1:** Demographic and professional characteristics, Brazil, 2020 (n = 403).

Characteristics	n	%
Sex		
Male	168	41.7
Female	235	58.3
Age (yrs)		
Under 30	27	6.7
31–40	101	25.1
41–50	99	24.5
51–60	114	28.3
Age >60 yrs	62	15.4
Professional experience (yrs)		
Under 10	84	20.8
11–20	106	26.3
21–30	104	25.8
Over 30	109	27
Works in public sector		
Exclusively	31	7.7
Mostly	193	47.9
Sometimes	51	12.6
Rarely	14	3.5
Never	114	28.3

**Table 2:** Responses to medical litigation, Brazil, 2020 (n = 403).

Question	n	%
Professional indemnity insurance		
Yes	165	40.9
No	238	59.1
Litigation/lawsuit against obstetrics versus other medical specialties		
Higher risk	381	94.5
Lower risk	01	0.2
No difference	18	4.5
I have no opinion	03	0.7
Recent impression of the frequency of lawsuits against obstetricians		
Increased	348	86.3
Decreased	02	0.5
Remained unchanged	36	8.9
I have no opinion	17	4.2
High settlement rates incentivize increased lawsuits against obstetricians		
Yes	344	85.3
No	38	9.4
I have no opinion	21	5.2
Settlement values in lawsuits against obstetricians		
High	298	73.9
Low	03	0.7
Reasonable	14	3.5
I have no opinion	88	21.8
Distinction between “adverse event” “medical error” and “professional malpractice” by the judicial system		
Never	26	6.4
Rarely	159	39.4
Sometimes	162	40.2
Often	41	10.2
Always	03	0.7
I have no opinion	12	3.0
Specialized medical courts to conduct lawsuits against healthcare professionals		
Yes	360	89.3
No	25	6.2
I have no opinion	18	4.4
Sued for “medical error” or knows an obstetrician who has been sued		
Yes	325	80.6
No	78	19.4

**Table 3:** Responses to defensive medicine in obstetrics, Brazil, 2020 (n = 403).

Question	n	%
Defensive medicine due to being sued or knowing someone who was sued		
Yes	298	73.9
No	69	17.1
Never been or known anyone who has been sued for “alleged medical error”	36	8.9
Avoids patients with high-risk pregnancies due to fear of professional litigation/lawsuit		
Never	204	50.6
Rarely	61	15.1
Sometimes	54	13.3
Often	41	10.1
Always	12	3.0
Does not practice obstetrics	31	7.7
In case of adverse event/complication, which option is at a higher risk of resulting in lawsuit		
Natural delivery	272	67.5
Cesarean section	02	0.5
Birth method irrelevant	125	31.0
Unlikely, regardless of the birth method	03	0.7
I have no opinion	01	0.2
Cesarean section without medical indication due to the threat of a lawsuit		
Yes	147	36.5
No	240	59.5
I do not practice obstetrics	16	3.9
Cesarean section due to fear of complications during normal delivery to avoid a lawsuit		
Yes	252	62.5
No	136	33.7
I do not practice obstetrics	15	3.7
Frequency of vaginal delivery by vacuum extractor or forceps, if indicated		
Never	97	24.1
Rarely	157	38.9
Sometimes	75	18.6
Often	28	6.9
Always	20	5.0
I do not practice obstetrics	26	6.5
Performed cesarean section as opposed to vaginal delivery assisted by vacuum extractor or forceps, when indicated, due to fear of a medical lawsuit		
Yes	205	50.9
No	145	36.0
I never perform vacuum extractor or forceps-assisted vaginal delivery	35	8.7
I do not practice obstetrics	18	4.5

Age, sex, or workplace (public or private) does not have a statistically significant influence on the chances of GOs having already been sued or knows a colleague that has either been sued or is performing defensive CS. The length of professional experience is the only demographic data that influences these chances in a statistically significant way. Doctors with 11 to 30 years of professional experience are more likely to have been sued or knows a colleague that has been sued when compared to both physicians with less experience and those with more professional experience. GOs with up to 30 years of professional experience are also more likely to performing defensive CS.

The results showed that obstetricians who had been sued or who knew a colleague that had been sued exhibited a significantly higher likelihood of performing defensive CSs (OR, 2.11; CI, 1.22–3.65) than physicians who had not been sued or did not know physicians who had been sued (Table 4). Professionals with at least 20 years of experience performed more defensive CS out of fear of litigation for potential adverse events of natural delivery than those with less experience. No statistically significant relationship was found between sex and defensive CS (Table 4).

**Table 4:** Risk factors by demographic, professional, and litigation-related characteristics leading obstetricians to perform defensive cesarean sections.

Variable	Odds ratio (CI 95%)	*P*-value^∗^
Has been sued or knows someone who has been sued		
Performed cesarean sections due to fear of complications from normal delivery to avoid litigation	2.11 (1.22–3.65)	<.01
Performed cesarean sections as an alternative to operative vaginal deliveries due to fear of complications that could result in a lawsuit against the physician	1.64 (0.90–2.99)	.086
Performed cesarean section due to fear of complications in normal delivery to avoid litigation		
Male	0.98 (0.63–1.53)	.915
Up to 20 years of professional experience	1.68 (1.08–2.63)	.02
Perception of a higher risk of lawsuits against obstetricians		
Performed cesarean sections due to fear of complications from normal deliveries to avoid litigation	6.07 (1.79–26.35)	<.01

The perception of a higher risk of lawsuits against obstetricians influenced the practice of DM and led to a more than 6-fold increase in CSs in specialists with this perception (OR, 6.07; CI, 1.79–26.35) compared to specialists who did not believe the presence of an increased risk of litigation in obstetrics existed; this difference was statistically significant (*P* < .01). Sufficient sample evidence was not available to validate the use of CS as an alternative to vacuum extractor or forceps-assisted vaginal births due to possible complications that could result in a lawsuit against the physician who has been sued or knew a colleague who has been sued (Table 4).

Although some Brazilian obstetricians responded that they avoid high-risk patients, their proportion was lower than that in the USA, and the difference was statistically significant.^[17]^ However, most obstetricians responded that they avoid performing vacuum extractor or forceps-assisted vaginal delivery, and the frequency of this procedure among Brazilian physicians was statistically lower than that performed by physicians in Turkey (Table 5).^[34]^

**Table 5:** Comparison between defensive medical attitudes in different countries.

Variable	Frequency	Countrytotal n (%)	Countrytotal n (%)	*P*-value^∗^
Avoids patients with high-risk pregnancies to avoid lawsuits	Never and rarely	Brazil372265 (71.2)	USA (16)18728 (15)	<.001
Performs vacuum extractor or orceps-assisted delivery	Often and always	Brazil37748 (12.7)	Turkey (32)10854 (50)	<.001

## 4 Discussion

The results of this study showed that most Brazilian obstetricians surveyed stated that they perform CS as a form of DM. Moreover, they believe that obstetrics is associated with a higher risk of lawsuits, thus making fear of litigation a factor that directly influences the performance of defensive CS.

The demographic and professional characteristics of the present sample were comparable to Brazilian medical demography data.^[44]^ The mean national age of GOs is 49.6 years, with a standard deviation of 12.3 years, and the mean age of this sample fell within the standard deviation. In Brazil, most GOs are women (56.6%); the same is true in this survey. Regarding the workplace, 73.1% of Brazilian physicians work in both public and private sectors,^[44,45]^ and in this study, 71.7% of the participants work in both public and private sectors.

The GOs perceive obstetrics as having a higher risk of litigation than other specialties and believed that the number of cases of litigations has increased in recent years. In Turkey, GOs also considered obstetrics as the highest-risk specialty, and like Brazil, they believed that the number of cases had increased.^[34]^

This perception of risk is related to DM practice, which can be defined as the conduct adopted by healthcare professionals based on the fear of litigation, as opposed to patient benefit^[24–26]^; therefore, it is neither an evidence-based medical practice nor in accordance with the *lege artis* principle. In Brazil, this perception of a higher risk of litigation has statistically increased the chances of performing defensive CS among obstetricians, which represents health risks to both women and children.^[4,5]^ Therefore, performing medical procedures such as CS as a purely defensive measure and without medical indication likely constitutes a violation of bioethical principles. The European Federation of Physicians, the American Board of Internal Medicine, and the American College of Physicians have defined these principles with the understanding that patients’ interests must be superior to medical interests. The fundamental principle is the primacy of patient well-being, autonomy, and social justice.^[46]^

Another issue obstetricians reported was lawsuit settlement amounts, which were deemed to be unreasonably high and were perceived to stimulate the judicialization of unfavorable outcomes. Studies have concluded that high settlements levied against obstetricians for alleged negligence/malpractice during vaginal delivery are correlated with increased CS, thereby suggesting that lower settlements may reduce CS rates.^[47,48]^

In cases of litigation against physicians, the respondents did not believe that the judicial system could adequately distinguish an “adverse event” from a “medical error.” Therefore, most GOs would support the formation of specialized courts to hear cases against healthcare professionals. Physicians outside Brazil noted the perception that courts cannot distinguish between terms such as “complication” and “medical error.”^[34,49]^ Similarly, GOs support specific laws for specialized medical courts.^[32]^ Therefore, these specific services would improve the judicial system and result in an increasingly rapid and accurate conclusion of lawsuits.^[34]^

Adverse events are responsible for a high number of deaths^[50]^; thus, an understanding of patient safety is important. To this end, the World Health Organization has published the International Classification for Patient Safety to standardize related concepts. Patient safety is defined as the reduction of unnecessary risks associated with healthcare, and adverse events are incidents that affect patients by causing harm, although they are not always the result of a medical error.^[51]^

The scientific community has discussed interventions by the legal system in medical disputes. Schifrin et al concluded that the legal system's conduct concerning lawsuits against physicians is questionable, and they found no evidence that the system complies with the fundamental precepts of repairing damages suffered or preventing further damages in the future.^[52]^

Legal models that deal with medical errors entail the conventional adversarial process, which seeks to find a culprit, or the no-fault system; this asserts the citizen's right to be compensated for damages that occur during medical care regardless of error, which is guaranteed by economic funding from professional contributions or taxes. New Zealand uses the no-fault system, wherein patients with insurance-covered comprehensive damage are compensated but not allowed to seek recourse in the judicial system.^[53,54]^ Japan and the state of Virginia in the USA have another type of no-fault system, which is exclusive to children born with severe neurological damage sustained during childbirth. In addition to reducing medical litigation, this model has helped improve perinatal quality and safety through the distribution of scientific materials based on data collected in such cases.^[55,56]^ Countries adopting the no-fault system to evaluate medical practices have lower CS rates than countries that use the conventional adversarial system.^[57]^ MacLennan suggested that the no-fault system may potentially prevent DM and could reduce unnecessary CS rates by 7%.^[58]^

In the current study, most GOs reported having been sued or knowing professionals who were sued. Most assumed that being sued in the medical profession or knowing someone who has been sued increased the defensiveness of their medical conduct; for example, this involved performing a defensive CS, which was statistically validated in the present study results.

Being sued may have other effects, such as decreased psychological well-being among physicians and increased rates of depression, anxiety, suicidal tendencies and burnout syndrome.^[59,60]^ In addition to these emotional consequences, GOs are concerned about humiliation^[59]^ and feared negative media exposure (traditional and social media), which subsequently influences the practice of DM and increases defensive CS rates.^[59,61–63]^

Some studies have suggested that these lawsuits are unnecessarily adversarial and damaging to both patients and physicians, thereby concluding that changes in the judicial system are required.^[64]^ This suggests that alternative measures to investigate alleged medical errors may reduce DM practice.^[63]^

Compared with GOs in other countries, GOs generally do not avoid attending high-risk pregnant women, but avoid performing vaginal delivery assisted by vacuum extractor or forceps.^[17,34]^ This situation can be attributed to the obstetricians’ perception that vaginal births are associated with a higher risk of lawsuits when complications do occur than CS. This observation has been reported by other researchers, and the risk of being sued is higher in cases of vaginal delivery that result in neurological sequelae, neonatal death, shoulder dystocia, vaginal birth after CS, and operative vaginal delivery.^[65,66]^

Lawsuits involving CS are usually based on the supposed delay in performing the procedure for patients in labor and not on the inability to perform the surgery.^[65]^ By contrast, Jena et al demonstrated that when physicians increase their CS rates, they experience a lower risk of being sued over time.^[67]^ Therefore, it is evident that the preference to avoid litigation by performing CS when the outcome of vaginal delivery is questionable.^[68]^ Social norms often view CS as a “modern” procedure that demonstrates better quality healthcare and fewer risks than vaginal delivery. The concern regarding possible damages from vaginal birth enables CS to be viewed as a preventive measure against adverse outcomes for both women and babies.^[69–71]^ In contrast, this false sense of security may be attributed to the lack of understanding that many CS-related complications are only observed at a later stage and that society is unable to correlate them to CS. For example, CS increases the risk of *placenta accreta spectrum*, *abruptio placentae*, *placenta previa*, and pelvic adhesions among women.^[72,73]^ Children born via CS have an increased risk of childhood asthma, and women who have a CS history are at an increased risk of unexplained stillbirths in subsequent pregnancies.^[4,74]^ This risk is not related to CS indication and is independent of previous obstetric complications and maternal characteristics.^[74]^

The results of this study show that obstetricians extensively perform defensive CS from fear of litigation arising from complications of vaginal delivery. This defensive attitude is reported in several other countries. Fear of litigation can vary between countries depending on the legal system. Dutch doctors have fewer legal complications when compared to other countries in Europe and, consequently, defensive CS are performed in a smaller quantity.^[75]^ Usually in countries where plural democracy predominates, such as America, Oceania and Europe, the legal system tends to favor the practice of defensive medicine.^[23,76]^ However, defensive medicine is also causing concerns in many other regions of the world,^[21,34,77,78]^ including countries with different political regimes than plural democracies. The Islamic Republic of Iran, for example, in addition to having a high rate of CS,^[79]^ also has a high frequency of DM,^[80]^ and obstetric litigation in this country reduces the tendency of doctors to perform vaginal deliveries.^[81]^ In Romania, physicians have stated that they are influenced by the risk of malpractice and thus perform more defensive CS.^[33]^ In Israel, concerns about legal claims of damage during childbirth influenced the increased frequency of physicians performing CS, even in the absence of clear medical indication.^[26]^ CS rates are also increasing in Italy and Germany partly due to defensive CS.^[82,83]^

Schifrin et al stated that obstetricians are being held hostage to the fear of litigation, leading to changes in medical practice. The rates of previous procedures that favored vaginal delivery have declined due to the emergence of lawsuits arising from adverse events, leading to unjustifiable increases in CS rates and the rise of ethical conflicts.^[52]^ However, performing defensive CS has been the reaction of obstetricians to the high burden of lawsuits arising from adverse events during vaginal births, and some authors believe that this attitude may be plausible and even rational, given the circumstances.^[48]^ However, practicing DM procedures without medical indications may be a violation of the principles proposed in the Universal Declaration on Bioethics and Human Rights, as it does not maximize the benefits of medical practice or minimize the harmful effects of these interventions, unless it is a clear and informed choice by the woman.^[84]^

The results generated by our research are consistent with the legal system based on the guilty model in force in Brazil. Having analyzed the responses to the questionnaire, we suggest that lawsuits influence the subsequent behavior of Brazilian GOs. Professionals have the perception that litigation rates have increased, which reflects the reality in Brazil. In addition, the GOs found the lawsuit settlement amounts to be high and stated that they do not trust that judges can distinguish between “adverse event,” “medical error” and “professional malpractice”. Therefore, they support the creation of specialized courts to hear cases against healthcare professionals. Most GOs (73.9%) had some kind of experience with legal actions, influencing their behavior in relation to the choice of delivery method; 67.5% affirm that the risk of legal proceedings during normal birth is higher, while only 0.5% consider CS more likely to result in litigation. Various factors can cause fear of litigation and subsequently favor defensive behaviors such as defensive CS, increasing their already high rates and exposing patients to unnecessary risks.

CS rates may decrease when society adopts an increasingly positive attitude toward vaginal delivery and understands that vaginal delivery has less adverse consequences than CS. This may happen if evidence-based medicine clearly confirms this claim. Programs that promote health must consider the social and cultural aspects that are crucial to healthcare. The judicial system is a part of this social context; however, it must incorporate measures to reduce the pressure litigation has on increasing rates of defensive CS.^[85]^

### 4.1 Strengths and limitations

Increasing CS rates are a challenge for healthcare managers in Brazil, and the varying causes of this problem must be understood to determine the optimal solution. This study, which is unprecedented in Brazil, highlights another potential cause for analysis. The validity of this study is reinforced by a reliable sample that is compatible with demographic medical data due to the sufficient sample size.

A possible limitation of this study was that the sample was not random; thus, the questionnaire was most likely answered by the GOs most interested in the topic, which may be those who had experienced or knew someone who experienced litigation. However, although the answers to the questionnaire were anonymous, some of the interviewees may not have admitted that certain procedures were performed for their own self-protection and not based on the patient's needs.^[76]^ Although some respondents did not report that their medical practices were self-protective, the numbers reported in the survey sufficiently demonstrated the presence of DM in obstetrics in Brazil. Another limitation is the inability to globalize the findings of this research, since defensive medicine varies according to the legal system of each country.^[75]^ However, the concerns with defensive medicine exists in almost all continents, suggesting that the subject is of international interest, and not only in Brazil.

## 5 Conclusion

Most GOs reported that obstetrics is associated with a higher risk of litigation and stated that they have either been sued or knows a colleague who has been sued. The majority of Brazilian GOs perform defensive CS. DM is caused by the fear of litigation,^[24–26]^ and because vaginal delivery increases the risk of litigation,^[65,66]^ professionals opt for defensive CS due to its lower risk of litigation.^[65,67]^ Furthermore, CS rates are lower in countries where judicial systems are not based on a guilty or not guilty system,^[56]^ demonstrating that alternative means to investigate adverse events may be appropriate^[63,64]^; in addition to reducing DM practice, litigations can be concluded more rapidly and accurately.^[34,63]^ This study showed that of all the GOs surveyed, those who perceived an elevated risk of obstetric litigation, had as well as having undergone previous lawsuits, or knew someone who was sued had influence on their medical acts by increasing the practice of DM increased the likelihood of performing defensive CS. DM is not the only cause of an increase in CS rates. However, we recommend that the results of this research on defensive CS be considered in the development of the next public health policy in Brazil in order to reduce the high rates of CS.

## **A**cknowledgments

We would like to thank Editage (www.editage.com) for English language editing and H0consultoria (www.h0consultoria.com) for their statistical services.

## Author contributions

**Conceptualization:** Edson Luciano Rudey, Maria do Carmo Leal, Guilhermina Rego.

**Data curation:** Edson Luciano Rudey.

**Formal analysis:** Edson Luciano Rudey.

**Funding acquisition:** Edson Luciano Rudey.

**Investigation:** Edson Luciano Rudey.

**Methodology:** Edson Luciano Rudey, Maria do Carmo Leal, Guilhermina Rego.

**Project administration:** Edson Luciano Rudey, Maria do Carmo Leal, Guilhermina Rego.

**Resources:** Edson Luciano Rudey.

**Software:** Edson Luciano Rudey.

**Supervision:** Edson Luciano Rudey, Maria do Carmo Leal, Guilhermina Rego.

**Validation:** Edson Luciano Rudey.

**Visualization:** Edson Luciano Rudey, Maria do Carmo Leal, Guilhermina Rego.

**Writing – original draft:** Edson Luciano Rudey.

**Writing – review & editing:** Edson Luciano Rudey, Maria do Carmo Leal, Guilhermina Rego.

## Supplementary Material

Supplemental Digital Content
