# Supplementary material for: Defensive medicine and cesarean sections in Brazil
Source: Medicine (Baltimore). 2021 Jan 8;100(1):e24176. doi: 10.1097/MD.0000000000024176 (PMC7793425; doi:10.1097/MD.0000000000024176)
Supplement: Supplemental Digital Content [file medi-100-e24176-s001.doc]

**Supplementary File 1 – Defensive medicine questionnaire for gynecologists and obstetricians**

| **Section 1: Personal data** | | |
| --- | --- | --- |
| Number | Question | Answer |
| 01 | How old are you? | __________ years old. |
| 02 | What is your gender identity? | ( ) Female  ( ) Male  ( ) Other |
| 03 | In which year did you graduate? | ____________ |
| 04 | Do you work in the public sector (SUS)? | ( ) No  ( ) Rarely  ( ) Sometimes  ( ) Often  ( ) Exclusively |
| 05 | Do you have professional liability insurance? | ( ) Yes  ( ) No |

| Section 2: **Personal feelings about litigation against obstetricians** | | |
| --- | --- | --- |
| Number | Question | Answer |
| 06 | How much do you perceive the risk of litigation/proceedings against professionals in the obstetrics specialty as compared to other areas? | ( ) Higher risk  ( ) Lower risk  ( ) No difference  ( ) I have no opinion |
| 07 | Do you think there has been an increase in lawsuits against obstetricians in recent years? | ( ) Yes  ( ) No  ( ) I have no opinion |
| 08 | Do you think that high rates of compensation alone can stimulate an increase in lawsuits against obstetricians? | ( ) Yes  ( ) No  ( ) I have no opinion |
| 9 | How much do you perceive the settlement amounts in lawsuits against obstetricians? | ( ) High  ( ) Low  ( ) Reasonable  ( ) I have no opinion |
| 10 | Do you think the justice system can distinguish between “adverse event,” “medical error,” and “professional malpractice”? | ( ) Yes  ( ) No  ( ) I have no opinion |
| 11 | Would you support the creation of specialized medical courts to hear cases against health professionals? | ( ) Yes  ( ) No  ( ) I have no opinion |

| **Section 3: Defensive medicine and cesarean section** | | |
| --- | --- | --- |
| Number | Question | Answer |
| 12 | How often do you avoid working with obstetrics to escape possible litigation/professional proceedings? | ( ) Never  ( ) Rarely  ( ) Sometimes  ( ) Often  ( ) Always  ( ) I do not practice obstetrics for other reason(s) not related to the risk of litigation |
| 13 | If an adverse event/complication occurs, do you think it is more likely that the doctor will be prosecuted when he/she assists in a normal delivery or when he/she performs a cesarean section? | ( ) Normal birth  ( ) Cesarean  ( ) Birth method not relevant  ( ) I have no opinion |
| 14 | Have you ever performed a cesarean section with no medical indication due to threat of a lawsuit from patients, relatives or third parties? | ( ) Yes  ( ) No  ( ) I do not practice obstetrics |
| 15 | Have you ever performed a cesarean section due to fear of complications from a normal childbirth to avoid a possible lawsuit? | ( ) Yes  ( ) No  ( ) I do not practice obstetrics |
| 16 | How often do you perform vacuum- or forceps-assisted vaginal delivery when indicated? | ( ) Never  ( ) Rarely  ( ) Sometimes  ( ) Often  ( ) Always  ( ) I do not practice obstetrics |
| 17 | Have you ever performed a cesarean section as opposed to vaginal delivery assisted by vacuum extractors or forceps, when indicated, for fear of complications that could generate a lawsuit against the doctor? | ( ) Yes  ( ) No  ( ) I never perform vaginal birth assisted by vacuum extractors or forceps  ( ) I do not practice obstetrics |
